# Descriptive Epidemiology of Objectively Measured Walking Among US Pregnant Women: National Health and Nutrition Examination Survey, 2005–2006

**DOI:** 10.5888/pcd12.150437

**Published:** 2015-12-10

**Authors:** Youngdeok Kim, Eunhee Chung

**Affiliations:** Author Affiliation: Eunhee Chung, Department of Kinesiology and Sport Management, Texas Tech University, Lubbock, Texas.

## Abstract

The objective of this study was to examine population-based prevalence of walking in the United States among pregnant women. Objectively measured walking data on 197 pregnant women who participated in the National Health and Nutrition Examination Survey 2005–2006 were analyzed. In general, pregnant women showed a level of walking below the recommendation; most walking was at low-intensity levels. These findings suggest that walking, particularly at higher intensity than usual, should be promoted among pregnant women.

## Objective

Women with normal pregnancies are recommended to engage in regular physical activity for maternal health benefits (1). Walking is a safe, effective, and accessible form of physical activity for pregnant women (2) and is a primary physical activity choice in this population (3). Despite its popularity, few attempts have been made to describe naturally occurring walking in pregnant women. Therefore, the objective of this study was to examine the prevalence of objectively measured walking in pregnant women by using the most recent population-based accelerometer data from the National Health and Nutrition Examination Survey, 2005–2006.

## Methods

We analyzed data from 197 pregnant women aged 18 to 45 years whose pregnancy was confirmed by laboratory test in a mobile exam center (MEC) during the National Health and Nutrition Examination Survey (NHANES), 2005–2006 (wwwn.cdc.gov/nchs/nhanes/search/nhanes05_06.aspx) and who had valid data on study variables, including 1 or more valid days of accelerometer data (mean, 4.3 days; standard error [SE], 0.2 days) (4). During NHANES 2005–2006, all ambulatory participants who participated in the MEC exam were eligible to wear the ActiGraph accelerometer (model 7164, ActiGraph) on their waist during waking hours for 7 consecutive days. The accelerometer data were screened to determine nonwear time, defined as 60 consecutive minutes of no activity or up to 2 consecutive minutes with activity counts less than 100. A valid day was defined as having 10 or more hours of wear time (mean, 13.3 h/d; SE, 0.2 h/d). Step count data were censored by excluding the steps featuring low intensity levels (<500 activity counts/min) (4) because the ActiGraph accelerometer likely records low-force movements as steps, which are not comparable to well-accepted pedometer scales (4). The outcome variables of interest were total steps per day (average total step counts accumulated during accelerometer wear time across valid days), aerobic steps per day (average step counts accumulated for ≥10 consecutive minutes during accelerometer wear time across valid days), and 1-minute peak cadence (highest steps/min across valid days). In addition, total minutes and steps accumulated across incremental walking intensity levels were examined by using cadence bands (0, 1–19, 20–39, 40–59, 60–79, 80–99, 100–119, and ≥120 steps/min) (5). Descriptors of each cadence band were modified from Tudor-Lock et al (4) to reflect the use of censored step counts data in this study: 0 = nonstepping activities; 1–19 = incidental steps; 20–39 = sporadic steps; 40–59 = purposeful steps; 60–79 = slow walking; 80–99 = medium walking; 100–119 = brisk walking; and ≥120 = faster locomotion. 

Outcome variables were summarized and compared across demographic variables obtained during the NHANES household and MEC interviews. The walking intensity levels described by cadence bands were compared across the stages (3 trimesters) of pregnancy. All analyses accounted for complex sampling design of NHANES by using survey procedures in SAS version 9.4 (SAS Institute).

## Results

On average, pregnant women took 5,245.8 (SE, 260.2) steps per day, which included 1,515.0 (SE, 189.6) aerobic steps per day and a peak cadence of 100.9 (SE, 1.5) steps per minute (Table 1). Total steps per day did not vary statistically by demographic variables with the exception of the stage of pregnancy. Pregnant women in the first trimester showed significantly higher mean total steps per day (mean, 5,586.8 steps/d; SE, 339.5 steps/d) compared with pregnant women in the third trimester (mean, 4,489.6 steps/d; SE, 308.1 steps/d). Pregnant women in the third trimester also showed significantly lower 1-minute peak cadence (mean, 94.9 steps/min; SE, 1.4 steps/min) compared with those in the first trimester (mean, 104.9 steps/min; SE, 2.1 steps/min) and second trimester (mean, 103.5 steps/min; SE, 3.1 steps/min).

**Table 1:** Accelerometer-Based Walking Parameters in Pregnant Women (n = 197)^a^, NHANES 2005–2006

Characteristic	% (SE)	Total Steps/Day, Mean (SE)^b^^,^ c	Aerobic Steps/Day, Mean (SE)^b^^,^ c ^,^ d	1-Minute Peak Cadence, Mean (SE)^b^^,^ e
**Total**	—	5,245.8 (260.2)	1,515.0 (189.6)	100.9 (1.5)
**Age, y**				
18–30	79.8 (2.8)	4,855.8 (195.3)	1,136.3 (85.9)	99.9 (1.4)
31–45	20.2 (2.8)	6,810.5 (1,107.4)	3,034.4 (1,054.3)	104.8 (4.5)
**Pregnancy trimester**				
1st	21.4 (5.7)	5,586.8 (339.5)^f^	1,215.4 (177.9)	104.9 (2.1)^g^
2nd	44.7 (5.6)	5,698.2 (555.7)	2,005.5 (481.6)	103.5 (3.1)^h^
3rd	33.9 (6.3)	4,489.6 (308.1)	1,084.7 (161.0)	94.9 (1.4)
**Race/ethnicity**				
Non-Hispanic white	60.4 (5.9)	5,205.8 (250.4)	1,287.2 (166.8)	102.1 (1.8)
Mexican American	20.1 (4.2)	4,752.4 (609.0)	1,545.3 (337.4)	99.3 (1.7)
Other	19.5 (4.2)	5,874.3 (1,067.4)	2,177.0 (1,024.3)	98.8 (2.6)
**Education**				
≤High school diploma	32.9 (4.4)	5,927.7 (434.4)	1,863.0 (405.2)	97.0 (2.3)
>High school diploma	67.1 (4.4)	4,881.8 (344.8)	1,329.2 (177.1)	102.8 (2.1)
**Marital status**				
Married/couple	84.3 (4.3)	5,385.4 (353.1)	1,618.5 (245.0)	101.6 (1.8)
Single or other	15.7 (4.3)	4,526.8 (473.9)	981.6 (387.8)	97.1 (2.6)
**Smoking status**				
Yes	5.2 (1.3)	8,356.2 (2,825.5)	5,149.8 (2,919.9)	99.2 (4.3)
No	94.9 (1.3)	5,065.0 (192.3)	1,303.7 (110.4)	101.0 (1.5)
**Family income-to-poverty ratio**				
1st tertile	22.1 (2.6)	5,897.5 (664.0)	2,318.5 (608.1)	97.8 (2.0)
2nd tertile	37.6 (3.9)	5,016.1 (230.5)	865.2 (137.6)	97.7 (1.7)^i^
3rd tertile	40.4 (3.8)	5,085.3 (392.3)	1,683.2 (212.3)	105.6 (2.7)
**Obese (1 year ago)^j^**				
Yes (BMI ≥30 kg/m^2^)	87.0 (4.6)	4,534.7 (349.2)	1,513.5 (190.6)	98.8 (1.4)
No (BMI <30 kg/m^2^)	13.0 (4.6)	5,341.8 (269.2)	1,526.5 (406.3)	101.2 (1.8)
**Previous live births^k^**				
Yes (≥1 live birth)	57.9 (5.7)	5,492.6 (370.2)	1,285.1 (158.4)	98.4 (2.1)
No (no live birth)	42.1 (5.7)	4,910.9 (250.2)	1,684.4 (403.6)	104.3 (1.7)

Abbreviations: BMI, body mass index; — , not applicable; NHANES, National Health and Nutrition Examination Survey; SE, standard error.

a The final sample represented 53.7% of total women with a positive pregnancy test in the NHANES 2005–2006 (n = 367) (wwwn.cdc.gov/nchs/nhanes/search/nhanes05_06.aspx) after excluding women who 1) were younger than 18 (n = 16); 2) did not participate in accelerometer data collection (n = 60); 3) did not provide valid accelerometer data (n = 59); and 4) did not provide valid responses on the stage of pregnancy (n = 30) and other study variables (n = 5). The final analytic sample was more likely to have higher education levels (*P* < .001) and less likely to be single (*P* = .03) and pre-obese (*P* < .001) than the final sample.

b All values accounted for the complex sampling design of NHANES 2005–2006. The general linear model was used to test group mean differences across the levels of categorical variables after controlling for accelerometer wear time.

c Least-squares mean step counts adjusted for accelerometer wear time.

d Average step counts accumulated for at least 10 consecutive minutes during accelerometer wear time across valid days.

e The highest steps/min during accelerometer wear time across valid days.

f Significantly different from third trimester (*P* = .02).

g Significantly different from third trimester (*P* = .004).

h Significantly different from third trimester (*P* = .02).

i Significantly different from third tertile (*P* = .01).

j BMI was calculated by using objectively measured current height (cm) and self-reported weight (kg) 1 year ago was used to determine obesity status (BMI ≥30kg/m^2^).

k Self-reported number of deliveries resulting in a live birth was used.

On average for the total sample, the largest portion of total steps per day was accumulated by sporadic walking (mean, 1,583.9 steps/d; SE, 84.3 steps/d), followed by purposeful walking (mean, 1,273.1 steps/d; SE, 55.9 steps/d) (Table 2). There were no remarkable differences in accumulated cadence patterns by stage of pregnancy. Although not significant, accumulated total steps per day tended to decline by stage of pregnancy at cadence levels less than 80 steps per minute. Pregnant women in the second trimester tended to have the highest prevalence of walking at cadence levels at or higher than 80 steps per minute.

**Table 2:** Accumulated Time and Steps per Day in Cadence Bands by Stage of Pregnancy (n = 197)^a^

Time and Steps	Cadence Band (Steps/Min)^b^							
	0	1–19	20–39	40–59	60–79	80–99	100–119	≥120
**Minutes/d**								
Total (SE)	646.2(4.6)	36.0 (2.0)	54.4 (2.9)	26.6 (1.2)	10.0 (0.3)	5.9 (0.4)	6.2 (2.2)	0.7 (0.5)
% (SE)	82.2 (0.6)	4.6 (0.3)	6.9 (0.4)	3.4 (0.2)	1.3 (0.1)	0.8 (0.1)	0.9 (0.3)	0.1 (0.1)
1st Trimester	635.2 (13.5)	41.2 (4.5)	56.0 (7.2)	28.4 (3.1)	12.0 (0.6)	6.4 (0.8)	6.6 (1.6)	0.2 (0.1)
% (SE)	80.6 (1.6)	5.1 (0.6)	7.3 (0.9)	3.7 (0.4)	1.5 (0.1)	0.8 (0.1)	0.9 (0.2)	0.02 (0.01)
2nd Trimester	644.1 (6.4)	34.8 (4.0)	52.8 (1.9)	27.1 (1.6)	10.1 (0.7)	6.6 (0.7)	9.0 (4.4)	1.4 (0.9)
% (SE)	82.0 (0.9)	4.6 (0.4)	6.6 (0.2)	3.4 (0.2)	1.3 (0.1)	0.8 (0.1)	1.3 (0.7)	0.2 (0.13)
3rd Trimester	655.2 (11.4)	34.6 (2.8)	55.3 (7.3)	25.0 (2.1)	8.6 (0.6)	4.7 (0.6)	2.4 (0.9)	0.1 (0.04)
% (SE)	83.5 (1.4)	4.2 (0.3)	7.0 (0.9)	3.2 (0.3)	1.1 (0.1)	0.6 (0.1)	0.3 (0.2)	0.01 (0.01)
*P* value^c^	.50	.46	.80	.55	.005	.06	.16	.43
**Steps/d**								
Total	—	428.3 (23.9)	1,583.9 (84.3)	1,273.1 (55.9)	683.5 (18.9)	525.1 (39.8)	661.9 (236.0)	90.2 (59.3)
% (SE)	—	9.3 (0.5)	32.7 (1.0)	25.3 (0.9)	13.4 (0.4)	9.5 (0.5)	8.5 (1.3)	1.3 (0.8)
1st Trimester	—	479.0 (58.8)	1,623.7 (204.6)	1,355.9 (148.4)	823.7 (40.6)	574.1 (74.3)	711.4 (171.6)	19.1 (9.3)
% (SE)	—	9.8 (0.8)	31.2 (1.4)	25.0 (0.8)	15.0 (0.9)	9.7 (0.8)	9.0 (1.7)	0.3 (0.1)
2nd Trimester	—	412.7 (40.3)	1,549.6 (59.1)	1,296.3 (75.2)	693.2 (47.6)	591.8 (72.8)	966.7 (468.7)	187.8 (130.8)
% (SE)	—	9.2 (1.0)	31.4 (1.7)	24.9 (1.5)	12.5 (0.5)	9.4 (0.7)	9.9 (2.2)	2.7 (1.7)
3rd Trimester	—	417.9 (42.2)	1,603.0 (206.4)	1,196.2 (102.8)	589.8 (38.2)	414.3 (54.2)	257.3 (105.4)	11.1 (4.5)
% (SE)	—	9.2 (0.5)	35.2 (1.6)	26.0 (0.8)	13.5 (0.9)	9.6 (0.9)	6.4 (1.0)	0.2 (0.1)
*P* value^c^	—	.57	.87	.55	.004	.06	.16	.43

Abbreviations: SE, standard error; —, not applicable.

a All values are presented as mean (SE) after accounting for complex sampling design of National Health and Nutrition Examination Survey 2005–2006 (wwwn.cdc.gov/nchs/nhanes/search/nhanes05_06.aspx) and were estimated using the censored minute-by-minute step counts data adjusted for accelerometer wear time.

b Descriptors of each cadence band were modified from Tudor-Lock et al (4) to reflect the use of censored step counts data in this study: 0 = nonstepping activities; 1–19 = incidental steps; 20–39 = sporadic steps; 40–59 = purposeful steps; 60–79 = slow walking; 80–99 = medium walking; 100–119 = brisk walking; and ≥120 = faster locomotion.

c *P *values were estimated for main group effects from general linear models after controlling for accelerometer wear time.

## Discussion

To the best of our knowledge, this is the first study to describe naturally occurring walking among representative samples of US pregnant women. Pregnant women with no complications are recommended to engage in at least 150 minutes of moderate-intensity aerobic physical activity per week (approximately 30 minutes per day on most days of week) (1), which is approximately equivalent to 7,000 to 8,000 steps per day (6). As with findings from previous studies (3,7), however, our findings demonstrated that pregnant women had low levels of physical activity, an average of 5,281.8 total steps per day, which falls below the recommendation. Total volume of walking and the highest level of walking attempts (1-min peak cadence) significantly declined as pregnancy progressed. Furthermore, our findings regarding walking intensities based on incremental cadence bands showed that pregnant women preferred to walk at low-intensity levels and rarely engaged in walking at moderate or vigorous intensity levels (≥100 steps/min) (6). This finding aligns with that of a previous study showing a low prevalence of vigorous-intensity physical activity among pregnant women (7).

The decline of physical activity throughout pregnancy may result from pregnancy-related physical changes. However, given the increased risk of maternal physical complications as pregnancy progresses (8,9) and the potential health benefits of physical activity to reduce such risks (10,11), pregnant women should be physically active during pregnancy. Furthermore, given recent findings showing the beneficial association of intensity of walking pace with health outcomes (eg, reduced risk of gestational diabetes mellitus) (12), pregnant women should be encouraged to engage in more walking activities and at higher intensity levels than usual.

Limitations to our study are the use of cross-sectional data and the validity of the waist-worn ActiGraph accelerometer to measure steps in pregnant women. Despite such limitations, our findings contributed additional information to the existing literature by focusing on objectively measured walking among a nationally representative sample of US pregnant women. Future studies are needed to examine the longitudinal patterns of walking in pregnant women to identify the modifiable influencing factors that can be addressed when developing future intervention strategies.
